# Long-Term Predictors of Hospitalized Reinfarction after an Incident Acute Myocardial Infarction

**DOI:** 10.3390/life12122090

**Published:** 2022-12-13

**Authors:** Timo Schmitz, Eva Harmel, Margit Heier, Annette Peters, Jakob Linseisen, Christa Meisinger

**Affiliations:** 1 Epidemiology, Medical Faculty, University of Augsburg, 86159 Augsburg, Germany; 2 Department of Cardiology, University Hospital of Augsburg, 86156 Augsburg, Germany; 3 KORA Study Centre, University Hospital of Augsburg, 86156 Augsburg, Germany; 4 Institute of Epidemiology, Helmholtz Zentrum München, 85764 München, Germany; 5 Institute for Medical Information Processing, Biometry and Epidemiology, Medical Faculty, Ludwig-Maximilians-Universität München, 80539 München, Germany; 6 German Center for Diabetes Research (DZD), 85764 Neuherberg, Germany

**Keywords:** myocardial infarction, reinfarction, population-based registry

## Abstract

The aim of this study was to compare characteristics of incident acute myocardial infarction (AMI) and first and second time reinfarctions in terms of sociodemographic characteristics, comorbidities, symptoms, treatment, clinical characteristics, medication and outcome. A further aim was to identify predictors for an increased risk of hospitalized reinfarction. Between 2000 and 2017, a total of 13,276 AMI cases were recorded by a population-based registry in the area of Augsburg, Germany, and were included in this study (11,871 incident events, 1217 cases of first-time reinfarction and 202 cases of second-time reinfarction). Median follow-up time was 5.3 years. For differences in baseline characteristics, Chi-square tests and analysis of variance (ANOVA) were calculated. To determine factors that are associated with an increased risk of hospitalized reinfarction COX regression models were fitted. Myocardial reinfarctions differ from incident events in some major characteristics such as the frequency of comorbidities, laboratory values, ECG presentation and therapy, but not regarding 28-day mortality. Moreover, typical comorbidities and risk factors (diabetes, hypertension, hyperlipidemia, smoking, impaired renal function) are associated with an increased risk of hospitalized reinfarction. Conversely, STEMI ECG, being married, German nationality and bypass surgery are predictors for a lower risk of hospitalized reinfarction. Incident AMI and reinfarction are distinctly different in many characteristics, which physicians should have in mind when treating patients with prior AMI. Typical comorbidities are risk factors for hospitalized reinfarction. This underlines the importance of comprehensive treatment of these comorbidities including education of patients and encouragement towards lifestyle adjustments.

## 1. Introduction

Acute myocardial infarction (AMI) is mainly derived from acute plaque rupture of atherosclerotic plaques in the coronary arteries. Therefore, the aim of acute invasive treatment (percutaneous coronary intervention = PCI) is restoral of blood flow and minimization of permanent damage, i.e., development of scar tissue. Since the underlying pathomechanism of atherosclerosis is not addressed by primary PCI, reduction of cardiovascular risk factors, with both optimal pharmacotherapy and lifestyle changes, seem to be of major importance for further disease management. One of the most important aims in this regard is the prevention of myocardial reinfarctions, which represent one of the major long-term complications.

Several prior studies examined characteristics and factors that are associated with an increased risk of reinfarction after AMI [1,2,3,4,5,6,7,8,9], hospital readmission after AMI [10,11,12,13] or requirement of re-interventions after discharge [14]. Nevertheless, most studies had observational times of one year or less. As reinfarctions can occur decades after a first-time event, studies with longer observational periods are required in order to get the full picture of characteristics and factors associated with higher risks of reinfarction. Moreover, most studies only examined certain AMI subgroups, e.g., patients with PCI intervention; studies based on complete myocardial infarction registers are scarce. Hence, in this study we compared important characteristics of AMI cases such as symptoms, treatment and comorbidities between first-time AMI and reinfarctions. Furthermore, we examined predictors that are significantly associated with the long-term risk of hospitalization due to reinfarction after an incident AMI.

## 2. Methods

### 2.1. Data Collection

The underlying data for this research was collected by the population-based Augsburg Myocardial Infarction Registry. It was established in 1984 as a part of the MONICA-project (Monitoring Trends and Determinants in Cardiovascular disease) and since then operated as the KORA Myocardial Infarction Registry [15]. The study area consists of the city of Augsburg, Germany and the two adjacent counties with a total of approximately 680,000 inhabitants. All cases of AMI were recorded on condition the patient survived longer than 24 h after hospital admission, was age 25 to 74 years (from 2000 until 2008) or 25 to 84 years (from 2009 until 2017) and had their primary residence within the study area. Trained study nurses conducted personal interviews using a standardized questionnaire during the hospital stay. Further data was collected elaborating the patient’s medical files. In this way, wide-ranging data for each case of AMI was collected including sociodemographic characteristics, risk factors, comorbidities, diagnostic procedures and treatment. More detailed information on data collection is available in previous publications [16,17].

The study has been approved by the ethics committee of the Bavarian Medical Association (Bayerische Landesärztekammer) and was performed in accordance with the Declaration of Helsinki. All study participants have given written informed consent.

For the analysis, all consecutive cases between January 2000 and December 2017 were considered. Cases of patients with reinfarction in that time frame, but incident AMI before 2000, were excluded. A patient was considered having had a hospitalized reinfarction when a further AMI after the incident AMI was recorded for this patient by the registry until the end of 2017. This approach was chosen due to the complete recording of all AMI cases in our population-based registry. Early reinfarctions within the first 28 days after the initial event were not considered as the registry did not record them as separate AMI cases.

Preexisting comorbidities and risk factors such as diabetes, hyperlipidemia and hypertension as well as smoking status and typical chest pain symptoms at the event were determined during the interview and validated by chart review if possible. For diabetes, it was not distinguished between type 1 and type 2 diabetes. Prehospital time was defined as the time period between onset of symptoms and hospital admission. Admission ECG was evaluated by physicians and each case was assigned to one of the following four groups: ST-elevation myocardial infarction (STEMI), Non-ST-evaluation myocardial infarction (NSTEMI), bundle branch block (BBB) and ‘ECG unknown’ (in case no information on admission ECG was available).

Estimated glomerular filtration rate (eGFR) was calculated by admission creatinine levels according to the CKD-EPI formula. Four categories were defined: ‘normal renal function’ (eGFR ≥ 60 mL/min/1.73 m^2^), ‘slightly impaired renal function’ (eGFR between 30 and 59 mL/min/1.73 m^2^), ‘heavily impaired renal function’ (eGFR < 30 mL/min/1.73 m^2^) and ‘no information on renal function’ (values for creatinine levels were only available since 2005).

For left-ventricular ejection fraction (EF), three categories were built: ‘severely restricted left-ventricular EF’ (≤30%), ‘not severely restricted EF’ (>30%) and ‘no-information on left-ventricular EF’.

In regard to acute treatment, it was recorded whether the following methods were conducted in direct context with the AMI event (within the hospital stay): PCI, Bypass therapy, lysis therapy (yes/no).

One combined variable (yes/no) was created for all four evidence-based medications at discharge (EBM): antiplatelet agents, beta-blockers, angiotensin-converting enzyme inhibitors (ACEIs) or angiotensin-receptor blockers (ARBs) and statins. All four are considered standard therapy after AMI.

Several in-hospital complications were combined to one variable ‘any in-hospital complication’. It was set to yes, whenever the patients had one or more of the following complications during their hospital stay: cardiogenic shock, left heart decompensation, bradycardia (<50/min), in-hospital reinfarction, ventricular tachycardia and ventricular fibrillation.

Information for the variables ‘family status’, ‘employment status’ and ‘nationality’ were extracted from the routinely conducted interview with the patients. Family status was classified into the groups ‘married’, ‘not married’ and ‘no information on family status’. The categories for the employment status were ‘currently employed’, ‘currently not employed’, ‘never employed’ and ‘no information on employment’. The variable ‘nationality’ was dichotomized into ‘german’ and ‘not german’.

A 28-day case fatality after AMI was evaluated by checking the vital status of all registered persons on a regular basis. Death certificates were obtained from the local health departments.

### 2.2. Statistical Analysis

Characteristics of the events such as comorbidities, clinical characteristics, treatment, and 28-day fatality were cross-tabulated with the number of AMI (incident AMI, first time reinfarction, second time reinfarction). Categorical variables are presented as total number and percentages, continuous variables are described as median and interquartile range (IQR) or mean and standard deviation (SD). To determine differences, the Chi^2^ test for categorical variables and a one-way ANOVA (analysis of variance) for continuous variable were performed. For continuous variables, extreme outliers and implausible values were removed from the analyses. Extreme outliers were identified by visually evaluating the plots of the cook’s distance values. The Levene test was performed to check the assumption of equal variance. If the Levene test rejected the assumption, a one-way ANOVA without the assumption of equal variance was performed. The continuous variable peak CKMB, Troponin I at admission, hemoglobin at admission, peak CRP, days in intensive care and prehospital time in minutes had significant *p*-values in the ANOVA and therefore post hoc *t*-tests with Bonferroni adjustment were performed.

COX regression models were calculated in order to identify predictors of an increased risk of hospitalized reinfarction. For this analysis, only patients who survived at least 28 days after their incident events were included in the analysis. Potential risk factors and comorbidities suggested by prior studies were considered for inclusion as well as variables that we suspected to be useful to predict reinfarction. To avoid overfitting, the initial number of variables included in the model was limited to a maximum of 25 variables. There were high percentages of missing values for the numeric variables peak CKMB, Troponin I at admission, hemoglobin at admission, peak CRP, prehospital time and days in intensive care. These variables were planned to be included in the COX regression models. In order not to disregard all cases with missing values in the aforementioned variables, multiple imputation by chained equations was conducted. Since all variables for which imputation was performed were numerical variables, the imputation method was linear regression. The number of iterations was 5 and the number of created imputed data sets was 5 as well. The imputation process was performed with the MICE-package (R statistic software, version 4.1.0., MICE-package version 3.14.0, authors: van Buuren S, Groothuis-Oudshoorn K).

Afterwards, each COX regression model was performed for each of the 5 imputed data sets separately and the results of the models were pooled afterwards. The first COX model was calculated including the following covariables: sex, age, hypertension, diabetes, hyperlipidemia, smoking status, typical chest pain symptoms, left ventricular ejection fraction, eGFR, type of infarction (ECG), PCI, bypass therapy, lysis therapy, EBM at discharge, any in-hospital complication, peak CKMB levels, Troponin-I levels at admission, peak CRP levels, hemoglobin levels at admission, prehospital time, days in intensive care, working status, family status and nationality.

Using backwards elimination, covariables that did not make a significant contribution to the model were eliminated step-by-step. This algorithm was repeated until the final model (parsimonious model) contained only variables that made a significant contribution. Sex and age were forced to stay in the model at any time. Consequently, the parsimonious model was adjusted for the following covariates: sex, age, hypertension, diabetes, hyperlipidemia, smoking status, type of infarction (STEMI, NSTEMI, bundle branch block), eGFR group, bypass therapy, family status, nationality (german, not german). Proportional hazard assumption was visually checked by Kaplan–Meier-curves, scaled Schoenfeld residuals against time and Log(-log(survival)) curves. The assumptions were considered to be sufficiently fulfilled. Nevertheless, a test for correlation between Schoenfeld residuals and time was significant for age, bypass therapy and family status. Therefore, we calculated a further COX regression model with two different time periods as a sensitivity analysis (see supplementary material, Table S1). The first period included the time of 28 days until 3000 days after incident AMI. The second period began with more than 3000 days after the first AMI. In the time-split model, no more significant correlation between scaled Schoenfeld residuals and time existed for any of the variables or time periods. Since this study includes AMI cases from almost two decades and some diagnostic and therapeutic procedures have changed and improved noticeably within this time frame, we performed another sensitivity analysis by calculating the parsimonious COX regression model including only cases from 2010 until 2017.

## 3. Results

### 3.1. Comparison of Characteristics between Incident AMI and Reinfarction

A summary presentation of the results can be found in Table 1. There were no differences in sex distribution between the groups (incident AMI, reinfarctions) with almost three quarters men in all events. The mean age at the incident event (64.1 years) was significantly lower in comparison to the mean age at the first (66.4 years) and second reinfarction (66.5 years), respectively. The percentage of patients living alone was higher at reinfarctions than at the initial event. More patients were currently employed at the time of the first AMI compared to reinfarction and accordingly the percentage of patients currently not employed increased with the number of events.

The prevalence of major cardiovascular risk factors (hypertension, diabetes mellitus, hyperlipidemia) was significantly higher at the first and second reinfarction compared to the incident AMI. The percentage of patients who were current smokers decreased after the incident AMI and consequently the proportion of ex-smokers increased in the same way. No striking differences between the groups could be observed regarding the percentages of patients having typical chest pain symptoms (ranging between 70% to 80% of all cases). Prehospital time was the highest for incident cases (median time: 157 min) and decreased for the second and third AMI event (median: 134 min and 133.5 min).

Among all incident AMI cases, 36% were typical STEMI cases, while 52.2% were NSTEMI cases and 7.5% of the cases were classified as BBB. The percentage of STEMI cases significantly decreased to 20.7% for the first reinfarction and to 17.4% for the second reinfarction. Contrary to this trend, the number of cases classified as BBB increased from 7.5% to 12.5% and 18.9%, respectively. Regarding reduced ventricular ejection fraction and eGFR, the percentage of cases with missing information increased for the reinfarctions, so that no reliable statement about the frequency of occurrence was possible.

The laboratory values peak-CKMB, hemoglobin at admission, Troponin I at admission and peak CRP levels were significantly higher at the incident event than at the reinfarctions (see Table 1 and Table 2). There were no significant differences between the first and second reinfarction.

In regard to treatment, the percentage of PCI was almost identical for all three groups. Bypass surgery and lysis therapy, however, were more frequently performed at the incident event than at the reinfarction events. The number of patients with all four EBMs at discharge did not vary between the groups. The mean number of days in intensive care was significantly higher for the first event (3.5 days) compared to reinfarction events (2.9 and 2.8 days). Nevertheless, the short-term outcome (in the meaning of 28-day case fatality) was similar for all events with about 7% of patients not surviving the first 4 weeks after the event.

### 3.2. Predictors of Hospitalized Reinfarction

**Figure 1:**
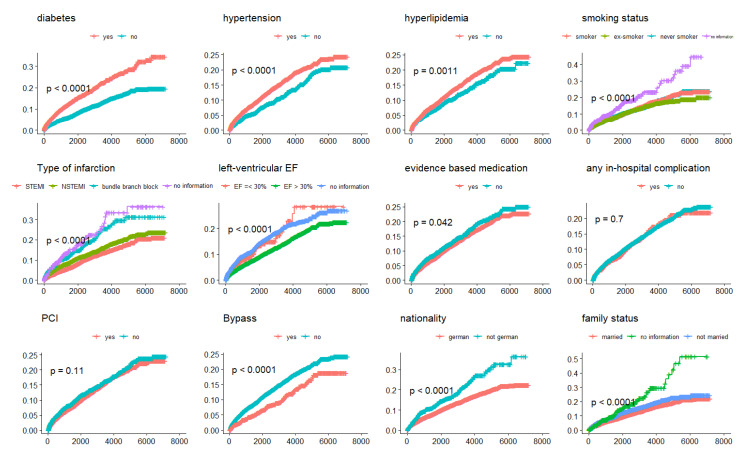
Kaplan–Meier curves visualizing cumulative hospitalized reinfarctions according to different characteristics. *p*-values are calculated by Log-rank tests.

A total of 10,052 patients with first-time AMI, who survived at least 28 days after the incident event and without missing values for relevant data, were included in the COX regression models. Of those, 7297 (72.6%) were men. Median follow-up time was 5.3 (IQR: 6.8) years. In the parsimonious model, older age was associated with an increased risk of hospitalized reinfarction. Likewise, the comorbidities and risk factors diabetes mellitus, hypertension, hyperlipidemia and current smoking were associated with an increased risk of hospitalized reinfarction as well (see Table 3 and Figure 1). STEMI ECG at admission and bypass surgery, however, were associated with a lower risk of hospitalized reinfarction. Furthermore, being married and of German nationality were significant predictors of a lower reinfarction risk. Figure 1 displays Kaplan–Meier curves of several predictors including the covariables of the parsimonious COX regression model.

The results of the COX regression model with the time-split function resembled the results of the model with only one time-period (see Supplementary Material, Table S1). Yet, the predictive values tended to be greater in the first time period than in the latter period. That implies that results are comparable between the two models, but the predictive values attenuate over time. In order to give an impression of the results of all initially considered variables that did not make it into the final model, in Table S2 of the supplementary material we present the results of the starting COX regression model including all initially considered variables.

Results from subgroup analysis including only cases from 2010 until 2017 were quite similar to the findings in the whole group. The results are displayed in the Supplementary Material, Table S3.

## 4. Discussion

Incident AMI and reinfarction were distinctly different in some major characteristics such as ECG representation, laboratory values and treatment such as bypass surgery and lysis therapy. First and second reinfarction, however, closely resembled each other in most characteristics. Although incident events seemed to be of higher severity, short-term mortality was not strikingly different from reinfarction.

The comorbidities and risk factors diabetes mellitus, hypertension, hyperlipidemia, impaired renal function and smoking as well as the sociodemographic factors ‘not married’ and ‘not german’ were independent predictors for an increased risk of reinfarction. However, ST-Elevation in the admission ECG and bypass therapy were associated with a significantly lower risk of reinfarction.

The comparison of important patient characteristics revealed some striking differences between incident AMI and reinfarction. First of all, prehospital time was significantly higher at the first AMI compared to further events. This appears very plausible, as patients gain a deeper understanding of their diseases after the first event and consequently have a higher awareness of specific symptoms. A review on causes of prehospital delay in myocardial infarction came to the conclusion that amongst other things, patients’ uncertainty about their symptoms and recognition of the importance of the symptoms play a major role in long prehospital delays [18]. In this study, the percentage of patients presenting with typical chest pain symptoms was similar at the incident event and at reinfarctions. Therefore, it seems conclusive, that not the clinical symptoms per se changed between first AMI and reinfarction, but that the perception of the symptoms was different and in this way lead to a decrease in prehospital time. This can be considered a very desirable adjustment as it is known that delay in treatment is associated with poorer outcomes and higher rates of in-hospital complications [19,20]. Nonetheless, prehospital time was not an independent predictor of reinfarction in the present study.

The prevalence of diagnosed cardiovascular comorbidities and risk factors such as hypertension, diabetes mellitus, and hyperlipidemia increased with the number of events, which fits to previously reported results [21]. It could be speculated, that this does not represent an actual increase in prevalence of important risk factors due to high number of unrecorded comorbidities prior to the first event. By thorough examinations following the first event, unknown comorbidities might be detected so that the percentage of diagnosed risk factors increases. An exception from this is the cardiovascular risk factor smoking. The number of current smokers decreases, and the number of ex-smokers increases after the first event, which is consistent with prior observations [22]. Smoking cessation is known to be a very effective secondary prevention measure after AMI [23]. Nevertheless, after the first reinfarction, no further decrease in current smokers can be observed in comparison to a second time reinfarction anymore.

The ECG presentation was very different at incident AMI and reinfarction. The percentage of STEMI decreased as the number of BBB increased with the number of infarctions. Prior infarction can lead to persisting ECG abnormalities such as BBB. This might interfere with new changes due to the current event and in this way hide typical ST-elevations in AMI. Another point to consider in regard to a lower number of STEMI’s at reinfarctions, is that reinfarctions might be less severe cases of AMI compared to incident events. This assumption can be drawn due to differences in laboratory values such as peak-CKMB, Troponin I at admission and peak CRP levels, which are all significantly higher at the incident event than in reinfarctions in the present investigation. Especially, CKMB levels are known to have a good correlation with myocardial damage and infarction size [24,25]. The assumption of a higher severity of incident events is also consistent with the longer stay at the intensive care unit compared to reinfarction cases. Nevertheless, average number of days at intensive care changed considerably over the period of study collection and in this way distorting the results. Further to be mentioned with regard to time at intensive care unit are suspected differences between STEMI and NSTEMI cases. Since the percentage of STEMI cases was significantly higher in incident cases, this might have affected the results as well.

A major explanation for the higher severity of first-time AMI might be a higher awareness and an enhanced willingness to seek medical help in case of a reinfarction. This could cause the effect that milder cases of reinfarction are more likely to be recognized and captured by the attending physicians. As well as the patients, physicians might also have a higher awareness of another AMI after a patient already had an incident AMI.

Rapid revascularization of the occluded coronary arteries is crucial in the treatment of AMI. Prior studies have shown that early reperfusion correlates with lower mortality rates [26,27,28]. Currently, the most commonly used method for revascularization is PCI, which was performed in about two thirds of the cases included in this study, regardless of the AMI type (incident vs reinfarction). Coronary artery bypass surgery is another important type of revascularization therapy. Indications can be, amongst others, multivessel diseases and severely calcified coronary artery lesions, patients with diabetes or recurrent in-stent restenosis [29]. Interestingly, the frequency of bypass surgery dropped from 14% among incident cases to 6% among second time reinfarction cases. Here too, it must be considered, that PCI techniques improved markedly between the years 2000 and 2017 including extended technical possibilities. That enabled the minimally invasive PCI to replace invasive bypass surgery in some cases of the AMI and consequently changing the rates of PCI and bypass therapy over the years.

A similar picture emerges for lysis therapy, which was performed in 4.5% of incident AMI cases and in less than 1% of reinfarctions. It must be considered that cardiac catheterization and PCI began to be widely used in the early 2000’s. The widespread use of PCI replaced lysis therapy for AMI in many parts. Since data collection for this study began with the year 2000 and ended in 2017, the frequency of PCI increased over the period of time; to the contrary, the frequency of lysis therapy dropped substantially. This circumstance could also explain the great decrease in lysis therapy for reinfarctions, as they happened in later times than the incident events.

Somewhat surprisingly, when considering the greater severity of incident events, the 28-day case fatality was not strikingly different from that of reinfarctions. A possible explanation could be the younger age of people with an incident event. Moreover, overall health status of patients with reinfarction might be worse than the one of patients with incident AMI due to ongoing comorbidities and older age of the patients. Additionally, in many reinfarction patients, the coronary artery disease might have progressed since the initial event and that circumstance might have complicated revascularization therapy in the reinfarction event. The above-mentioned aspects may have contributed to the relatively high case fatality in reinfarctions.

### 4.1. Predictors of Hospitalized Reinfarction

In this study, we did not find significant differences between men and women, even though women tended to have a non-significantly lower risk of hospitalized reinfarction. Older age was significantly associated with an increased risk of reinfarction, although the effect size was very modest. A prior study from Sweden on a working-class population found no association between reinfarction and age or sex (follow-up time: one year) [1] For age they used categorical data, which restricts comparability.

The important comorbidities and risk factors diabetes, hypertension, hyperlipidemia and current smoking status were significant predictors of an increased risk for hospitalized reinfarction. This matches findings from prior studies, which also found associations between common cardiovascular comorbidities and risk factors such as diabetes and smoking and an increased risk of reinfarction [2]. As it could be expected, the predictive values of the mentioned factors attenuate over the course of time (see supplementary material). Somehow an exception from this is the presence of a diabetes disease, as it has the highest HR of all comorbidities and remains being a significant predictor in the latter time period as well. Prior studies demonstrated that diabetes is associated with a more diffuse and severe coronary artery disease in patients with AMI [30,31,32]. This by itself could explain a higher risk of reinfarction. Furthermore, it complicates recanalization therapy and, in this way affects the risk of a further AMI [33].

Another strong predictor for hospitalized reinfarction is kidney function represented by eGFR. A slightly or significantly impaired kidney function is significantly associated with a higher risk of hospitalized reinfarction. It has been shown in prior studies that moderate to severe renal impairment is associated with increased coronary artery atherosclerosis [34,35,36]. Both diseases share mutual risk factors such as hypertension, diabetes and hyperlipidemia and might be different manifestations of the same underlying pathophysiological processes. Nevertheless, it remains unclear to which extent the relationship is causal.

The type of infarction according to admission ECG presentation was also associated with the risk of reinfarction. In this study it was found that patients with STEMI had a lower risk of hospitalized reinfarction than patients with NSTEMI or bundle branch block during a median follow-up time of 5.3 years. This is in contrast to results from a prior study, where STEMI was associated with a higher risk of reinfarction in the first years after AMI [1]. It must be considered that ST-elevations are a very specific indication of AMI, which is why the acute medical handling and treatment differs considerably between STEMI and NSTEMI cases (faster and mostly prehospital diagnosis, more rapid revascularization therapy in form of PCI, etc.). This does not only improve the outcome of the event, but supposedly also causes an enhanced awareness of the severity of the disease, which might motivate the patient to implement important lifestyle changes such as smoking cessation or body weight reduction. The assumption of a potential association between treatment delay and reinfarction is supported by the results of a study that reported a significantly increased risk of reinfarction with a longer symptom onset-to-balloon time [2]. Nevertheless, in this study, prehospital time (as a major component of treatment delay) was excluded from the parsimonious model as it was not significantly associated with the risk of reinfarction.

Unexpectedly, PCI and lysis therapy at the incident event were not significantly associated with a lower risk of hospitalized reinfarction. Many studies proved that PCI is associated with a better short and long-term outcome after AMI [37,38,39]. This could suggest that PCI also leads to a lower risk of reinfarction. Nevertheless, there was no significant association. In about two-thirds of all AMI cases included in this study PCI was performed, so there was a minority of patients who did not receive coronary intervention. It can be assumed that these patients did not receive PCI due to either multimorbidity and high age, premature death or denial of invasive interventions by the patients. The first two points, directly or indirectly, lead to a higher short-term mortality and consequently making a reinfarction more unlikely or even impossible. This might partially explain the missing association between PCI and lower probability of reinfarction. To further investigate this issue, COX regression models with interaction terms were calculated (results not shown). No significant interaction between PCI and age was found. However, an interaction term between PCI and year of the event (2000 until 2017) revealed a significant interaction (*p*-value: 0.027). During the time period the rate of PCI increased from 36.7% in the year 2000 to 73.2% in the year 2017, which might have affected the overall results.

In contrast to treatment with PCI, bypass surgery was significantly associated with a lower risk of hospitalized reinfarction. In certain cases (e.g., patients with multivessel diseases, left main coronary artery stenosis), bypass therapy substantially improves the overall outcome (e.g., short- and long-term mortality) after AMI [40,41,42]. The results from this study suggest that bypass surgery is also very effective in preventing hospitalization due to reinfarction in AMI patients, which could be the consequence of a very effective and long-lasting revascularization. Nevertheless, it must be considered that only a non-representative minority of patients in this study (14.4% among patients with an incident AMI) underwent bypass surgery. Because no causality could be shown in this observational study, the results on associations between invasive treatment and risk of reinfarction must be interpreted very cautiously.

The sociodemographic parameters ‘nationality’ and ‘family status’ were significantly associated with the risk for hospitalized reinfarction. Patients who were not married and who were not of German nationality had an increased risk for hospitalized reinfarction, which is somehow contrary to prior results [1]. An explanation that comes to mind is a worse adaptation to the disease (e.g., medication, lifestyle changes), which might be caused, amongst other things, by communication problems, lower financial resources or a lack of social support.

### 4.2. Strengths and Limitations

This study is characterized by some strengths. First to mention is the high number of cases from a population-based registry with consecutive enrollment, which minimizes the risk of selection bias. The long observational period provides data on reinfarction happening several years after the incident AMI. In addition to information on the actual event, a large number of sociodemographic characteristics, risk factors, comorbidities and information on in-hospital complications and treatment was collected.

Nevertheless, there are some limitations to our study. Since only patients up to 74 years (2000 until 2008) and up to 85 years (2009 until 2017) were included, results can’t be applied to older patients. Due to the heterogeneous, population-based sample, the results and generic conclusion from this study might not be applicable to specific subgroups, e.g., specific age groups, patients treated with specific PCI techniques etc. Furthermore, reinfarction that lead to prehospital death or a fatal outcome within the first 24 h after admission were not captured by this study. In consequence, the study might lack a number of very severe reinfarctions, which might affect the results. Furthermore, the results may not be generalized to all ethnic groups since no information on ethnicity was available. Information on preexisting bundle branch block was not available as well. Moreover, we might not have considered all relevant confounders and cannot exclude possible reverse causation.

## 5. Conclusions

First-time AMI and reinfarction are distinctly different in many characteristics such as admission ECG presentation or laboratory values. The knowledge of such differences might help physicians in the diagnostic process and in therapy decision. This applies especially to AMI suspected cases of patients with a history of prior AMIs. This study further revealed that the well-known comorbidities/risk factors diabetes, hypertension, hyperlipidemia and smoking are not only predictors for CAD and AMI, but are also predictors for an increased risk of hospitalized reinfarction. This circumstance underlines the importance of comprehensive treatment of these risk factors also after an incident AMI. This includes education of the patients on the topic and encouragement towards a lifestyle adjustment (e.g., smoking cessation). The results of this study suggest that this is extraordinarily important for foreign patients with AMI, as those patients have a significantly increased risk of hospitalized reinfarction. A more or less existing language barrier could be suspected to play a major role in this regard. Therefore, it appears to be essential that all patients are able to develop an understanding of their disease and the underlying influencing factors.

## Figures and Tables

**Table 1:** Characteristics of incident AMI and first and second time reinfarction. Categorical variables are presented as total numbers (percentages) and continuous variables are presented as median (25th- and 75th quartile). *p*-values were calculated with Chi-Square tests for categorical variables and analysis of variance (ANOVA) for continuous variables.

	Incident AMI	First Reinfarction	Second Reinfarction	*p*-Value	
	*n* = 11,871	*n* = 1217	*n* = 202		*n*
male	8469 (71.4)	887 (72.9)	148 (73.6)	0.4299	13,276
age (mean, SD)	64.1 (11.4)	66.4 (11)	66.5 (10.3)	<0.0001	13,276
**Comorbidities**					
hypertension	9052 (76.3)	1103 (90.7)	187 (93)	<0.0001	13,276
diabetes mellitus	3729 (31.4)	582 (47.9)	106 (52.7)	<0.0001	13,276
hyperlipidemia	6909 (58.3)	906 (74.5)	160 (79.6)	<0.0001	13,276
**Smoking status**				<0.0001	13,276
current smoker	3760 (31.7)	275 (22.6)	42 (20.9)		
ex-smoker	3332 (28.1)	466 (38.3)	76 (37.8)		
never smoker	3403 (28.7)	348 (28.6)	57 (28.4)		
smoking status unknown	1364 (11.5)	127 (10.4)	26 (12.9)		
**Clinical characteristics**					
typical chest pain symptoms yes	9239 (77.9)	950 (78.1)	146 (72.6)	0.239	13,276
prehospital time in minutes	157.0 (83–495)	134.0 (77–316.5)	133.5 (75–284.75)	0.0043	9816
**Type of infarction (ECG)**				<0.0001	13,276
STEMI	4275 (36)	252 (20.7)	35 (17.4)		
NSTEMI	6194 (52.2)	751 (61.8)	113 (56.2)	-	
Bundle branch block	885 (7.5)	152 (12.5)	38 (18.9)	-	
ECG unknown	505 (4.3)	61 (5)	15 (7.5)	-	
**left ventricular EF**				<0.0001	13,276
≤30%	665 (5.6)	92 (7.6)	11 (5.5)		
>30%	8536 (72)	774 (63.7)	113 (56.2)		
no information on EF	2658 (22.4)	350 (28.8)	77 (38.3)		
any in-hospital complication yes	2183 (18.4)	225 (18.5)	43 (21.4)	0.5567	13,276
Kidney function				<0.0001	13,276
eGFR ≥ 60 mL/min/1.73 m^2^	6108 (51.5)	556 (45.7)	83 (41.3)		
eGFR 30–59 mL/min/1.73 m^2^	2377 (20)	360 (29.6)	59 (29.4)	-	
eGFR < 30 mL/min/1.73 m^2^	560 (4.7)	145 (11.9)	36 (17.9)	-	
eGFR unknown	2814 (23.7)	155 (12.7)	23 (11.4)	-	
days in intensive care				<0.0001	13,276
median (IQR)	2 (1–4)	2 (1–3)	2 (1–3)		
mean (SD)	3.5 (5.5)	2.9 (4.9)	2.8 (3.9)		
died within the first 28 days	782 (6.6)	92 (7.6)	15 (7.5)	0.3949	13,276
**Laboratory value**					
peak CKMB	56 (25–144)	42 (21–104)	42 (21–82)	<0.0001	11,493
Troponin I at admission	0.640 (0.13–4.07)	0.240 (0.06–1.12)	0.435 (0.12–2.2575)	<0.0001	7904
hemoglobin at admission	141 (129–152)	136 (120–148)	136 (118–148)	<0.0001	10,308
peak CRP	4.44 (1.21–13.855)	2.74 (0.77–11.57)	3.47 (0.785–8.93)	<0.0001	12,703
**Therapy**					
PCI yes	8040 (67.8)	810 (66.6)	132 (65.7)	0.8666	13,276
Bypass yes	1703 (14.4)	107 (8.8)	12 (6)	0	13,276
Lysis yes	531 (4.5)	9 (0.7)	1 (0.5)	0	13,276
EBM at discharge	7853 (66.2)	825 (67.8)	127 (63.2)	0.332	13,276
**Sociodemographic characteristics**					
**Family status**				0.0086	13,276
married	7920 (66.8)	760 (62.5)	124 (61.7)		
not married	3086 (26)	370 (30.4)	63 (31.3)	-	
family status unknown	853 (7.2)	86 (7.1)	14 (7)	-	
**Employment status**				<0.0001	13,276
currently employed	2478 (20.9)	141 (11.6)	25 (12.4)		
currently not employed	4982 (42)	544 (44.7)	98 (48.8)	-	
never employed	167 (1.4)	17 (1.4)	2 (1)	-	
no information on employment	4232 (35.7)	514 (42.3)	76 (37.8)	-	
nationality German yes	10,792 (91)	1073 (88.2)	182 (90.5)	0.0081	13,276

**Table 2:** Results of the post-hoc t-test with Bonferroni adjustment for continuous variables with significant *p*-values in the ANOVA.

Peak CKMB	Incident AMI	First Reinfarction
first reinfarction	<0.0001	-
second reinfarction	<0.0001	0.2
**Troponin I at admission**	incident AMI	first reinfarction
first reinfarction	0.0053	-
second reinfarction	0.7341	1
**hemoglobin at admission**	incident AMI	first reinfarction
first reinfarction	<0.0001	-
second reinfarction	<0.0001	1
**peak CRP**	incident AMI	first reinfarction
first reinfarction	<0.0001	-
second reinfarction	0.008	1
**days in intensive care**	incident AMI	first reinfarction
first reinfarction	0.00014	-
second reinfarction	0.10383	1
**prehospital time in minutes**	incident AMI	first reinfarction
first reinfarction	0.0017	-
second reinfarction	0.3482	1

**Table 3:** Results of the parsimonious COX regression model for hospitalized reinfarction after incident AMI.

Variable	Hazard Ratio [95% CI]	*p*-Value
**sex**		
male	1 (reference)	
female	0.92 [0.80–1.05]	0.226
**Age**	1.01 [1.00–1.02]	0.003
**hypertension**		
no	1 (reference)	
yes	1.19 [1.02–1.38]	0.024
**diabetes**		
no	1 (reference)	
yes	1.64 [1.45–1.54]	<0.001
**hyperlipidemia**		
not	1 (reference)	
yes	1.17 [1.03–1.33]	0.013
**smoking status**		
never smoker	1 (reference)	
current smoker	1.18 [1.01–1.38]	0.039
ex-smoker	0.92 [0.79–1.07]	0.291
no information	1.19 [0.91–1.54]	0.196
**Type of infarction (ECG)**		
STEMI	1 (reference)	
NSTEMI	1.25 [1.10–1.42]	<0.001
bundle branch block	1.76 [1.40–2.20]	<0.001
no information	1.88 [1.41–2.52]	<0.001
**eGFR**		
≥60 mL/min/1.73 m^2^	1 (reference)	
30–59 mL/min/1.73 m^2^	1.35 [1.13–1.61]	<0.001
<30 mL/min/1.73 m^2^	2.82 [2.14–3.71]	<0.001
no information	1.59 [1.39–1.82]	<0.001
**Bypass therapy**		
no	1 (reference)	
yes	0.59 [0.49–0.72]	<0.001
**family status**		
married	1 (reference)	
not married	1.24 [1.09–1.42]	0.001
no information	1.31 [0.99–1.75]	0.061
**nationality**		
German	1 (reference)	
not German	1.56 [1.30–1.87]	<0.001

## Data Availability

The data will not be shared. Due to restrictions from Helmholtz Zentrum München, data are available upon request for any researcher based on a standard agreement on data provision within the KORA Research Platform.

## Supplementary Materials

The following supporting information can be downloaded at: https://www.mdpi.com/article/10.3390/life12122090/s1, Table S1: Results of the parsimonious COX regression model for reinfarction after incident AMI with a time-split after 3000 days. Table S2: Results of the starting COX regression model including all initially considered variables. Table S3: Results of the parsimonious COX regression model for reinfarction after incident AMI including only cases from 2010 until 2017.
