# Supplementary material for: Long-Term Predictors of Hospitalized Reinfarction after an Incident Acute Myocardial Infarction
Source: Life (Basel). 2022 Dec 13;12(12):2090. doi: 10.3390/life12122090 (PMC9784794; doi:10.3390/life12122090)
Supplement: Supplementary file 1 [file life-12-02090-s001.zip › life-2019352-supplementary.pdf]

Supplementary material

Title: Long-term predictors of hospitalized reinfarction after an incident acute myocardial infarction

Timo Schmitz, Eva Harmel, Margit Heier, Annette Peters, Jakob Linseisen and Christa Meisinger

Table S1: Results of the parsimonious COX regression model for reinfarction after incident AMI with a time-split after 3000 days.

	Time period: 28 days until 3000 days		*> 3000 day*	
Variable	Hazard ratio [95% CI]	p-value	Hazard ratio [95% CI]	p-value
sex				
male	1 (reference)			
female	0.96 [0.83-1.11]	0.576	0.85 [0.58-1.25]	0.406
age	1.01 [1.00-1.02]	< 0.001	0.98 [0.97-1.00]	0.0998
hypertension				
no	1 (reference)		1 (reference)	
yes	1.26 [1.06-1.49]	0.008	0.97 [0.69-1.36]	0.863
diabetes				
no	1 (reference)		1 (reference)	
yes	1.62[1.42-1.84]	< 0.001	1.77 [1.29-2.42]	< 0.001
hyperlipidemia				
not	1 (reference)		1 (reference)	
yes	1.18 [1.03-1.36]	0.015	1.13 [0.80-1.58]	0.498
smoking status				
never smoker	1 (reference)		1 (reference)	
current smoker	1.27 [1.07-1.51]	0.006	0.80 [0.55-1.17]	0.218
ex-smoker	0.99 [0.84-1.18]	0.942	0.59 [0.39-0.89]	0.013
no information	1.22 [0.92-1.61]	0.176	1.15 [0.58-2.28]	0.685
Type of AMI				
STEMI	1 (reference)		1 (reference)	
NSTEMI	1.25 [1.09-1.44]	0.002	1.26 [0.91-1.73]	0.164
BBB	1.68 [1.33-2.15]	< 0.001	2.19 [1.17-4.09]	0.014
no information	1.79 [1.31-2.46]	< 0.001	2.34 [1.10-4.98]	0.027
eGFR (ml/min/1.73m²)				
≥ 60	1 (reference)		1 (reference)	
30-59	1.33 [1.10-1.60]	0.003	1.45 [0.74-2.84]	0.276
< 30	2.81 [2.12-3.72]	< 0.001	1.52 [0.21-11.14]	0.683
no information	1.61 [1.39-1.86]	< 0.001	1.54 [1.09-2.18]	0.016
Bypass therapy				
no	1 (reference)		1 (reference)	
yes	0.53 [0.41-0.65]	< 0.001	1.05 [0.70-1.59]	0.810
family status				
married	1 (reference)		1 (reference)	
not married	1.26 [1.09-1.46]	0.001	1.07 [0.74-1.55]	0.725
no information	1.21 [0.89-1.66]	0.230	2.14 [1.05-4.36]	0.035
nationality				
German	1 (reference)		1 (reference)	
not German	1.47 [1.21-1.80]	< 0.001	2.13 [1.37-3.132]	< 0.001

Table S2: Results of the starting COX regression model including all initially considered variables.

Variable	Hazard ratio [95% CI]	p-value
sex		
male	1 (reference)	
female	0.90 [0.78 - 1.04]	0.169
age	1.01 [0.99 - 1.02]	0.048
hypertension		
no	1 (reference)	
yes	1.20 [1.03 - 1.39]	0.021
diabetes		
no	1 (reference)	
yes	1.61 [1.43 - 1.82]	< 0.001
hyperlipidemia		
not	1 (reference)	
yes	1.19 [1.05 - 1.35]	0.007
smoking status		
never smoker	1 (reference)	
current smoker	1.18 [1.01 - 1.38]	0.042
ex-smoker	0.91 [0.78 - 1.06]	0.219
no information	1.14 [0.88 - 1.49]	0.322
Typical chest pain symptoms		
yes	1 (reference)	
no	0.92 [0.78 - 1.08]	0.314
no information	0.71 [0.29 - 1.73]	0.449
Prehospital delay (minutes)	1.00 [1.00 - 1.00]	0.599
Type of AMI		
STEMI	1 (reference)	
NSTEMI	1.19 [1.03 - 1.36]	0.017
BBB	1.63 [1.30 - 2.05]	<0.001
no information	1.78 [1.32 - 2.39]	<0.001
Left ventricular EF		
>30%	1 (reference)	
≤30%	1.30 [0.96 - 1.76]	0.097
no information	1.24 [1.09 - 1.41]	0.784
eGFR (ml/min/1.73m²)		
≥ 60	1 (reference)	
30-59	1.33 [1.11 - 1.59]	0.002
< 30	2.60 [1.94 - 3.47]	< 0.001
no information	1.48 [1.27 - 1.72]	< 0.001
Any in-hospital complication		
no	1 (reference)	
yes	0.99 [0.83 - 1.17]	0.871
Days in intensive care	1.01 [1.00 - 1.02]	0.166
Peak CKMB levels	1.00 [1.00 - 1.00]	0.359
Troponin-I levels at admission	1.00 [1.00 - 1.00]	0.647
Hemoglobin levels at admission	1.00 [1.00 - 1.00]	0.337
Peak CRP levels	1.00 [0.99 - 1.01]	0.896
PCI		
no	1 (reference)	
yes	0.92 [0.78 - 1.07]	0.274
Bypass therapy		
no	1 (reference)	
yes	0.53 [0.41 - 0.68]	< 0.001

Lysis therapy		
no	1 (reference)	
yes	0.86 [0.66 - 1.10]	0.230
All four evidence based medications at discharge		
yes	1 (reference)	
no	1.06 [0.93 - 1.22]	0.361
family status		
married	1 (reference)	
not married	1.24 [1.09 - 1.42]	0.001
no information	1.31 [0.98 - 1.75]	0.064
working status		
currently employed	1 (reference)	
currently not employed	1.09 [0.92 - 1.29]	0.308
never employed	1.11 [0.70 - 1.78]	0.651
no information	1.02 [0.84 - 1.23]	0.859
nationality		
German	1 (reference)	
not German	1.56 [1.30 - 1.87]	< 0.001

Table S3: Results of the parsimonious COX regression model for reinfarction after incident AMI including only cases from 2010 until 2017.

Variable	Hazard ratio [95% CI]	p-value
sex		
male	1 (reference)	
female	0.96 [0.75-1.21]	0.682
age	1.01 [1.00-1.02]	0.166
hypertension		
no	1 (reference)	
yes	1.32 [0.98-1.77]	0.067
diabetes		
no	1 (reference)	
yes	1.70[1.38-2.10]	< 0.001
hyperlipidemia		
not	1 (reference)	
yes	1.27 [1.03-1.57]	0.026
smoking status		
never smoker	1 (reference)	
current smoker	1.27 [0.96-1.71]	0.097
ex-smoker	0.98 [0.76-1.28]	0.901
no information	1.81 [1.07-3.09]	0.027
Type of AMI		
STEMI	1 (reference)	
NSTEMI	1.48 [1.17-1.87]	0.001
BBB	1.82 [1.25-2.65]	0.002
no information	1.43 [0.84-2.43]	0.19
eGFR (ml/min/1.73m²)		
≥ 60	1 (reference)	
30-59	1.45 [1.14-1.85]	0.003
< 30	2.53 [1.73-3.7]	< 0.001
no information	3.11 [1.78-5.42]	< 0.001
Bypass therapy		
no	1 (reference)	
yes	0.53 [0.37-0.76]	< 0.001
family status		
married	1 (reference)	
not married	1.16 [0.92-1.46]	0.211
no information	1.75 [0.95-3.21]	0.073
nationality		
German	1 (reference)	
not German	1.62 [1.19-2.22]	0.002

In another sensitivity analysis, the variable PCI treatment (yes/no) was additionally included into this model (table S3). Receiving a PCI treatment was associated with a non-significantly decreased risk of reinfarction with a hazard ration of 0.76 [0.58-1.00], p-value: 0.05297].
